# Emphysematous Cystitis: A Fatal Complication of Uncontrolled Type-2 Diabetes Mellitus

**DOI:** 10.7759/cureus.30028

**Published:** 2022-10-07

**Authors:** Darshankumar M Raval, Vaishnavi M Rathod, Nilay S Patel, Riya Dobariya, Bhavya Sharma

**Affiliations:** 1 Department of General Medicine, Sir Sayajirao General (SSG) Hospital and Medical College Baroda, Vadodara, IND

**Keywords:** uti, severe sepsis, complication of type-2 diabetes mellitus, uncontrolled diabetes mellitus, complicated urinary tract infection, emphysematous cystitis

## Abstract

Emphysematous cystitis (EC) is a rare type of complicated urinary tract infection mostly seen in elderly females with diabetes, characterized by gas within the bladder lumen and wall. The presenting symptoms are variable, ranging from no symptoms to severe sepsis. The commonly isolated organisms in urine cultures are *Escherichia coli* and *Klebsiella pneumoniae*. Imaging studies, namely plain conventional abdominal radiography and computed tomography, are necessary to make a definitive diagnosis of EC. The management includes medical treatment with culture-guided antibiotics, whereas surgical intervention such as cystectomy is rarely required in severe cases. Here, we have a case of a 48-year-old diabetic female diagnosed with EC on radio imaging. The patient was aggressively treated with higher antibiotics such as piperacillin/tazobactam, clindamycin, and fosfomycin along with measures to control blood sugars. However, she developed severe sepsis and succumbed to death. Our report presents one of the rare cases of EC as a life-threatening complication in diabetics, suggesting that every case of urinary tract infection in uncontrolled diabetics should be thoroughly investigated and treated to prevent fatal complications.

## Introduction

Emphysematous cystitis (EC) is a very uncommon type of complicated urinary tract infection (UTI), characterized by gas within the bladder lumen and wall. The usual presenting complaints are variable ranging from no symptoms to severe sepsis. EC is mainly seen in elderly females with uncontrolled diabetes mellitus. Urine cultures often isolate *Escherichia coli *and *Klebsiella pneumoniae*. Imaging studies, namely plain conventional abdominal radiography and computed tomography, are necessary to make a definitive diagnosis of EC [1]. The mortality rate of EC (7.4%) is lower than that of emphysematous pyelonephritis (EP), which is 24%; however, EC is a less common condition and, therefore, data on mortality from EC is still scarce [2]. The majority of the patients can be treated with a combination of bladder drainage, intravenous higher antibiotics, and insulin for glycemic control. Early medical treatment can help to achieve a good prognosis without requiring surgical intervention [1].

Here, we present the case of a 48-year-old diabetic female who presented with complaints of fever and hematuria and was diagnosed with EC on radio imaging. The patient was treated with higher antibiotics along with medications to control blood sugars; however, she developed severe sepsis and succumbed to death, suggesting EC is a life-threatening complication in diabetics. 

## Case presentation

A 48-year-old female who was a known case of hypertension and type-2 diabetes mellitus for 15 years and was on regular treatment with anti-hypertensive (amlodipine and atenolol), oral hypoglycemic agents (OHA) (metformin, glimepiride, and voglibose) and subcutaneous plain insulin, presented to our tertiary care hospital with chief complaints of low-grade fever, generalized weakness, and increased urinary frequency for past 15 days. The patient also had complaints of six to seven episodes of watery diarrhoea, nausea, vomiting, and hematuria for the last day. There was no complaint of burning micturition, flank pain, pain while micturition, abdominal pain, decreased urine output, cough, breathlessness, pedal oedema, facial puffiness, peri-orbital oedema, or bleeding from other sites. The general examination at the time of presentation to the hospital revealed normal temperature, pulse 90 per minute, Blood pressure (BP) 106/70 mmHg, oxygen saturation of 99% on room air on pulse oximetry, and random blood sugar (RBS) of 228 mg% with negative urinary ketones (excludes diabetic ketoacidosis (DKA)). The systemic examination was not significant for any abnormal findings, and symptoms/signs of uremia such as altered sensorium, altered sleep pattern, breathlessness, bilateral crepitation in lung fields, and extensor plantar reflexes were absent. A baseline electrocardiogram (ECG) was suggestive of left axis deviation (LAD) with left ventricular hypertrophy (LVH).

The initial routine blood investigations revealed sepsis with a high total count, raised C-reactive protein (CRP), high erythrocyte sedimentation rate (ESR), high blood sugars, and high lactate level on arterial blood gas analysis (ABGA). A urine routine examination was suggestive of UTI (Table 1). The provisional diagnosis of UTI-induced sepsis in uncontrolled diabetes was made and the patient was started on intravenous ceftriaxone and metronidazole along with subcutaneous plain insulin for the management of uncontrolled diabetes. Symptomatic treatment was also started in form of anti-pyretic, anti-emetics, and anti-diarrheal medications. Further investigations showed very high glycosylated haemoglobin (HbA1c), suggesting uncontrolled diabetes for a long duration (Table 2). Tests done to rule out other endemic and epidemic causes of fever were negative (Table 2).

**Table 1:** Serial Routine Investigations SGPT: Serum glutamic pyruvic transaminase; SGOT: serum glutamic-oxaloacetic transaminase; HCO3: bicarbonate; pCO2: partial pressure of carbon dioxide; pO2: partial pressure of oxygen

Investigations	Day 1	Day 3	Day 5	Day 7
Hemoglobin (gm/dL)	11.2	10.7	9.36	10.1
Total Count (per cumm)	26070	18600	14000	20400
Differential Count (N/L/E/M %)	78/20/01/01	70/28/01/01	66/30/02/02	84/12/02/02
Platelet Count (per cumm)	299000	287000	373000	213000
Urea (mg/dL)	21	43	58	48
Creatinine (mg/dL)	0.85	0.87	0.93	1.15
Bilirubin- Total/Direct/Indirect (gm/dL)	0.7/0.3/0.4	0.6/0.2/0.4	0.8/0.3/0.5	1.2/0.4/0.8
SGPT (U/L)	36	38	45	56
SGOT (U/L)	38	37	39	49
Alkaline Phosphate (IU/L)	112	110	124	167
Random Blood Sugar (mg/dL)	304	121	280	310
C-reactive protein (CRP) (mg/L)	69.8	70	92	92
Erythrocyte sedimentation rate (ESR) (mm/hour)	24	32	38	44
Serum sodium (mmol/L)	131	128	135	150
Serum potassium (mmol/L)	5.0	5.2	5.8	3.3
Urine Routine and Microbiology	Blood present. Turbid appearance. Protein trace. Pus cells – 8-10/high power field (h.p.f.). Red cells – 12-15/ h.p.f.	-	-	Blood +++ Turbid appearance. Protein Trace. Pus cells – >100 (plenty) h.p.f. Red cells – 15-20/ h.p.f.
Arterial Blood Gas Analysis (ABGA)	(On Room air)	(On Face Mask Oxygen)	(On noninvasive Ventilator)	(On noninvasive Ventilator)
pH-	7.418	7.22	7.38	7.3
pCO2 (mmHg)–	48.8	15.5	28.2	30.5
pO2 (mmHg)–	92	86	91	94
HCO3 (mmol/L)-	30.8	10.7	16.6	19.2
Lactate (mmol/L)–	2.61	1.2	1.0	3.5

**Table 2:** Other Investigations

Investigations	Result
Glycosylated hemoglobin (HbA1c)	11.5%
Human immunodeficiency virus (HIV) 1 & 2 antibodies	Negative
Hepatitis B surface antigen (HBsAg)	Negative
Hepatitis C virus (HCV)	Negative
Serum Widal	Negative
Dengue NS-1 antigen	Negative
Chikungunya IgM antibody	Negative
Malarial parasite	Not detected
Stool routine & microbiology	Normal

The blood, urine and stool culture were sent to isolate the causative organisms for sepsis (Table 3). The antibiotics were changed according to the culture reports and a fosfomycin sachet (3 grams) was given to treat UTI.

**Table 3:** Culture and Sensitivity Reports

Specimen	Organism Isolated	Drugs: Sensitive	Drugs: Resistant
Blood	*Klebsiella* Spp.	Ceftriaxone. ceftazidime. ceftazidime/ clavulinic acid. amikacin. levofloxacin. meropenum. piperacillin/tazobactam	-
Blood	Coagulase Negative Staphylococci	Amoxycillin/clavulinic acid. cefoxitin. gentamycin. erythromycin. clindamycin. co-trimoxazole. vancomycin. linezolid.	Penicillin.
Urine	Escherichia coli	Fosfomycin (200 microgm).	nitrofurantoin. nalidixic acid. cefotaxime. levofloxacin. co-trimoxazole. cefuroxime. doxycycline

**Figure 1:**
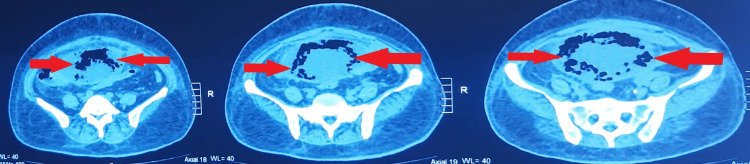
Axial section of Contrast-Enhanced Computed Tomography (CECT) Abdomen and Pelvis showing Emphysematous Cystitis

**Figure 2:**
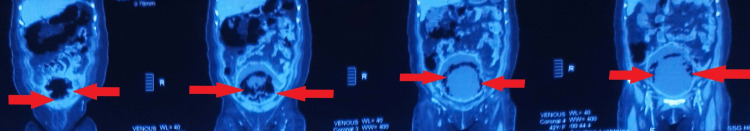
Coronal section of Contrast-Enhanced Computed Tomography (CECT) Abdomen and Pelvis showing Emphysematous Cystitis

**Figure 3:**
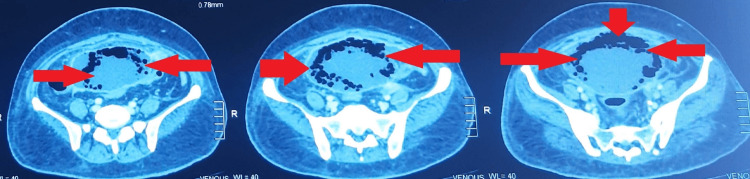
Axial section of Contrast-Enhanced Computed Tomography (CECT) Abdomen and Pelvis with contrast showing Emphysematous Cystitis

**Figure 4:**
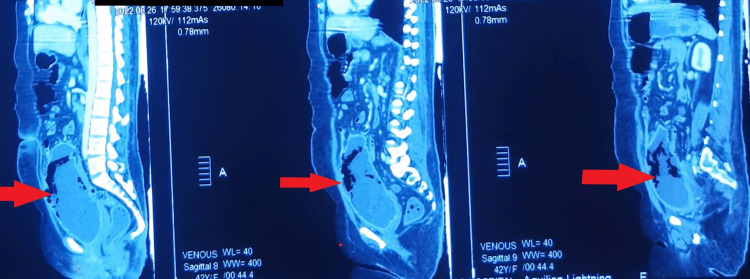
Sagittal Section of Contrast-Enhanced Computed Tomography (CECT) Abdomen and Pelvis showing Emphysematous Cystitis

Ultrasonography (USG) images of the kidneys were suggestive of changes of cystitis with moving echogenic foci; therefore, contrast-enhanced computed tomography (CECT) of the abdomen and pelvis was advised. CECT abdomen was suggestive of EC. (Table 4) (Figures 1, 2, 3, 4)

**Table 4:** Radiological Investigations

Investigation	Comments
Ultrasonography (USG) of Abdomen and Pelvis	Right Kidney: mild hydronephrosis (HN) with upper hydroureter (HU) of 6mm; Normal Bladder: wall appears irregular and thickened up to 7 mm, dense hyper echoic sludge and moving echogenic foci are seen. Possibility of cystitis.
Contrast Enhanced Computed Tomography (CECT) Abdomen and Pelvis	Urinary bladder appears distended and shows multiple air foci within and along its wall with surrounding severe fat stranding. Wall appears thickened and irregular measuring 4.2 mm. No calculus, mass, or diverticulum is seen. Suggestive of emphysematous cystitis. Both kidneys are normal. Rest of the scan is normal.

The patient responded initially to the treatment; however, even after stepping up the antibiotics to piperacillin/tazobactam and clindamycin, according to culture reports, she deteriorated after three to four days of treatment. Her blood sugars were difficult to control with subcutaneous insulin for which intravenous insulin drip with close blood glucose monitoring was started. The patient developed hypotension and hypoxia for which she was shifted to the intensive care unit (ICU) where inotropic support and mechanical ventilation were started. The urologist’s opinion was sought and bladder drainage was done as advised. However, after seven days of aggressive management, the patient succumbed to death.

## Discussion

EC is an uncommon type of complicated UTI characterized by the presence of gas in and around the wall of the urinary bladder. The gas is a product of the fermentation of bacterial or fungal infections in the urinary tract [3]. Irritative symptoms such as urinary urgency or frequency, burning micturition, discomfort in the abdomen, hematuria, or passage of air in urine (pneumaturia) are common presenting complaints [3]. Our patient also presented with symptoms of UTI such as fever and hematuria.

EC is more commonly seen in patients with diabetes, especially in females, making diabetes mellitus a major risk factor for the condition. Other factors predisposing to this condition are urinary tract outlet obstruction, chronic UTIs, neurogenic bladder, immune deficiency, and indwelling urethral catheters [4]. Our patient was a middle-aged female with a known case of diabetes and hypertension for 15 years and was on regular treatment. However, her diabetes was not under control by both OHA and insulin as evident by high HbA1c levels. Hence, in our patient, uncontrolled diabetes can be considered as a causative factor for EC.

Through laboratory investigations, our patient was found to have severe sepsis with a raised total count, ESR, CRP, lactate levels in the blood, and features of UTI on urine microscopy. The urine culture isolated *Escherichia Coli *as a causative organism. The literature also mentions *Escherichia coli *as the most common organism causing EC; however, other organisms are also isolated from the urine of EC patients such as *Klebsiella pneumonia*, *Enterobacter aerogenes*, *Staphylococcus aureus*, *Proteus mirabilis*, streptococci, *Candida albicans,* and *Clostridium perfringens* [3]. The imaging modalities are the major diagnostic tools for EC, where CT of the abdomen is superior to plain x-ray, by precisely defining the location and extent of collected gas in the urinary bladder. CT scan also distinguishes EC from emphysematous pyelonephritis, which has higher mortality compared to EC and requires urgent nephrectomy [5]. Our patient was diagnosed with EC by CECT abdomen where air foci were seen in and around the bladder wall. 

The majority of the patients are managed by medical treatment and only a few patients require surgical interventions such as debridement or cystectomy. Conservative management involves antibiotics according to culture and sensitivity reports, strict control of blood sugar, bladder drainage, intravenous fluid, and treatment of predisposing conditions. Antibiotics are given for two to three weeks [6]. Our patient was also started with higher antibiotics intravenously according to culture reports. She was also managed for high blood sugar by intravenous drip in ICU. She initially responded to the treatment; however, she later deteriorated. Bladder drainage was done as advised by the urologist. 

EC, in some patients, may be complicated as bladder dysfunction, rupture of the bladder wall, and obstruction of urination, leading to bilateral renal impairment, sepsis, and septic shock, as seen in our patient [7]. During the treatment, our patient went into hypotension and hypoxia despite adequate treatment and thus transferred to ICU where she expired even after aggressive medical treatment.

## Conclusions

EC is an uncommon type of UTI, commonly occurring in females with uncontrolled diabetes mellitus. Although it is a rare entity, its prognosis is overall better than emphysematous pyelonephritis. However, severe untreated infection can lead to sepsis, septic shock, and even death. The usual management includes medical therapy with culture-guided antibiotics and management of comorbid conditions, while surgical treatment is required in severe complicated cases. Here, we presented a case of EC in an uncontrolled diabetic female, who succumbed to death even after aggressive medical management. Timely diagnosis, appropriate management, and control of diabetes mellitus are essential to reduce morbidity and mortality in such cases; otherwise, EC can end as a lethal complication for diabetics.
